# Encyclopædia Medica

**Published:** 1900-03

**Authors:** 


**Encyclopædia Medica.** Under the General Editorship of Chalmers Watson, M.B. Vol. II.—*Brachial Plexus to Digestion.* Pp. vi., 562. Edinburgh: William Green & Sons. 1899.

This work, the first volume of which was reviewed in our last number, is on similar lines to its predecessor. The articles are rather more numerous, amounting to sixty-two, and those on the nervous system have a fair number of diagrams which are original and clear. Several articles on Brain extend to over a hundred pages, and are written by well-known authorities on the nervous system. The article on Bronchitis, extending to some twenty-two pages, is by Dr. Watson Williams, and a perusal of it leads one to the inference that there are more resources available than are commonly employed. Dr. S. H. Habershon gives a good summary of Bronchiectasis, and speaks favourably of the continuous inhalation of creasote, and of drainage when the evidence is in favour of a single cavity.

Dr. Urquhart remarks that "the neuroses incident to the change of life have not been studied with that care and accuracy which the subject demands," and accordingly he writes a very good article of six pages on Climacteric Insanity. We may mention incidentally that he considers it reasonable to suppose that the artificial injection of ovarian substance will in a measure restore the balance which has been destroyed by the production of an artificial menopause. The subject of Climate and Acclimatisation is well summed up by Dr. R. W. Felkin, of the British East Africa Company. Various articles, on such subjects as Colour Vision by Bickerton, Constipation by Langwill, Infantile Convulsions by Coutts, Diabetes Mellitus by Williamson, Diet by Hutchison, and Digestion by T. H. and J. A. Milroy, are all good summaries of our knowledge on the various subjects, and show that this volume will not be consulted in vain by those who desire the most modern views in various departments of medicine.

